# Characterization of a Complete Genome Sequence of Molluscum Contagiosum Virus from an Adult Woman in Australia

**DOI:** 10.1128/MRA.00939-20

**Published:** 2021-01-07

**Authors:** Subir Sarker, Sally R. Isberg, Ajani Athukorala, Ravi Mathew, Nolasco Capati, Md. Hakimul Haque, Karla J. Helbig

**Affiliations:** a Department of Physiology, Anatomy and Microbiology, School of Life Sciences, La Trobe University, Bundoora, VIC, Australia; b Centre for Crocodile Research, Noonamah, NT, Australia; c Oz Pathology, Millner, NT, Australia; d Top End Skin Cancer Care, Casuarina, NT, Australia; e Department of Veterinary and Animal Sciences, Faculty of Agriculture, Rajshahi University, Rajshahi, Bangladesh; KU Leuven

## Abstract

The complete genome sequence of molluscum contagiosum virus 1 (MOCV1) isolate NT2017 was sequenced from a tissue sample from an Australian woman. The genome consisted of 185,655 bp encoding 169 predicted open reading frames. Phylogenetically, isolate NT2017 was most closely related to an MOCV1 strain from Slovenia.

## ANNOUNCEMENT

Molluscum contagiosum virus (MOCV) belongs to the genus *Molluscipoxvirus*, in the family *Poxviridae*, and possesses a large double-stranded DNA genome of approximately 190 kbp and a G+C content of >60% (1). Early genomic studies suggested the existence of four major MOCV genotypes designated MOCV1 to MOCV4 (2) and several genotypic variants (3, 4). MOCV1 is the most prevalent genotype worldwide and causes cutaneous infections in the pediatric population, with only rare occurrences in adults (5). MOCV infection with mild symptoms has previously been reported in Australia (6, 7). Except for a single unpublished genome of MOCV (GenBank accession no. MH646551), there are no other genomes available for MOCVs detected in Australia. Here, we report the identification and genomic characterization of MOCV1 isolate NT2017.

The cutaneous tissue sample was collected from the left-hand side of the nose of a 39-year-old woman in 2017. Histologically MOCV-diagnosed formalin-fixed paraffin-embedded (FFPE) cutaneous tissue was aseptically sectioned, and total genomic DNA (gDNA) was extracted using a QIAamp DNA-FFPE tissue kit (Qiagen, USA). The library preparation was conducted with 1 ng of total gDNA using the Illumina Nextera XT DNA library prep kit, as reported previously (8, 9). The quality and quantity of the prepared library were assessed using an Agilent TapeStation system by the Genomic Platform, La Trobe University. Cluster generation and sequencing were performed using a MiSeq reagent kit version 3, generating 301-bp paired-end reads, as described previously (10, 11). The sequence data were processed using Geneious version 10.2.2 and CLC Genomics Workbench version 9.5.4 as previously described (12, 13). A total of 4,695,968 raw reads were preprocessed to remove adapters, ambiguous base calls, and poor-quality reads, followed by mapping against the human genome (Homo sapiens GRCh38, GenBank accession no. GCF_000001405.26) to remove host DNA contamination. Unmapped reads were used as input data for *de novo* assembly using the SPAdes assembler version 3.10.1 (14). This yielded a single contig of 185,602 bp. The resulting contig was further checked and validated by mapping back the trimmed reads. The draft genome sequence was further checked by mapping with a reference MOCV1 genome (GenBank accession no. U60315). This resulted in the generation of a 185,655-bp genome, including two inverted terminal repeats, each 2,505 bp long with a G+C content of 62%, and an average coverage of 404.20×. The MOCV1 genome was used as a reference genome for the annotation of the newly sequenced isolate using Geneious version 10.2.2, and further verification of the predicted open reading frames (ORFs) longer than 50 amino acids was performed using CLC Genomics Workbench version 9.5.4. Pairwise identity of the representative *Molluscipoxvirus* species against the MOCV1 isolate on the basis of complete genome nucleotide sequences was calculated using Base-by-Base software (15).

The MOCV1 isolate NT2017 genome contained 169 predicted methionine-initiated ORFs encoding proteins, which have been annotated as putative genes and were numbered from left to right. Comparative analysis of the protein sequences encoded by the predicted ORFs using BLASTX and BLASTP (16) identified homologs with significant protein sequence similarity to other MOCVs (E value, ≤e-5) for 168 ORFs, and only an open reading frame A (ORF-A) was found to be unique.

**FIG 1:**
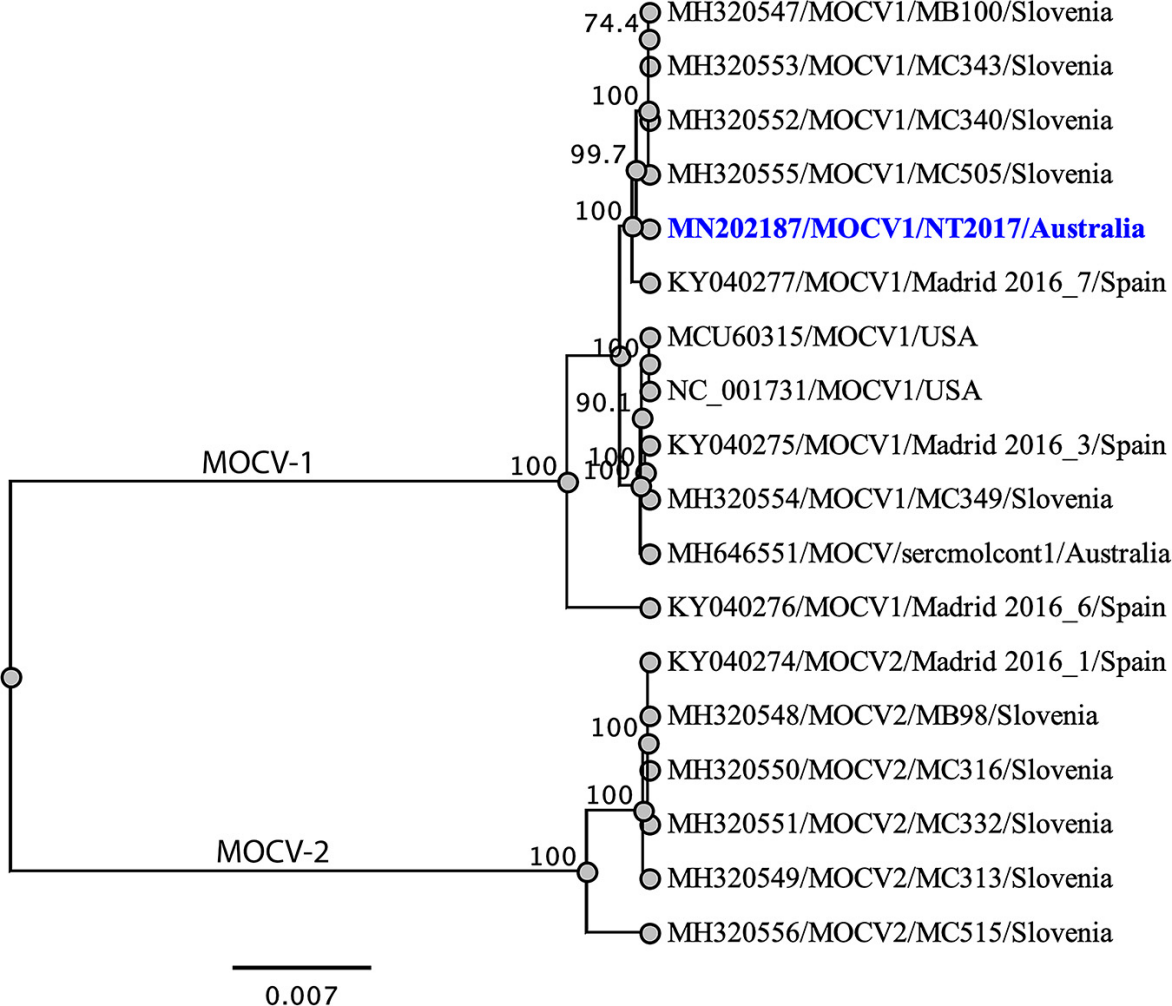
Phylogenetic relationship between MOCV isolated from Australia and other selected MOCV strains under the genus *Molluscipoxvirus*. The selected genomes were aligned with MAFTT version 7.450 using the G-INS-i (gap open penalty, 1.53; offset value, 0.123) algorithm implemented in Geneious version 10.2.2 (17). A maximum likelihood tree was constructed from the selected molluscipoxviruses using Geneious version 10.2.2. The numbers on the left show bootstrap values as percentages, and the MOCV1 isolate NT2017 is highlighted in blue. The labels at the branch tips refer to the original *Molluscipoxvirus* GenBank accession number followed by the abbreviated species name, isolate name, and country of isolation.

Phylogenetic analysis was conducted using selected full-genome sequences under the genus *Molluscipoxvirus*; NT2017 clustered in the same subclade as MOCV1 strains isolated from Slovenia (with nucleotide identities ranging from 99.86 to 99.87) with high bootstrap support (∼100%) (Fig. 1).

### Data availability.

This whole-genome sequence of MOCV1 isolate NT2017 has been deposited in DDBJ/ENA/GenBank under the accession no. MN202187. The version described in this paper is the first version, MN202187.1. The raw sequencing data from this study have been deposited in the NCBI Sequence Read Archive (SRA) under the accession no. SRR12569148 (BioProject no. PRJNA660887; BioSample accession no. SAMN15963002).
